# Historical Notes on Poisoning

**Published:** 1872-09

**Authors:** 


### SELECTED ARTICLES.

### *Historical Notes on Poisoning.*

The Professor of Forensic Medicine at King's College, London (David Farrier, M. A., M. D.,) delivered a Lecture with the above title, which is reported in the *British Medical Journal.* We extract the first part, and shall print the conclusion in our next:

The earliest use of poisons seem to have been for the purpose of anointing arrows; and the word which is used to denote poison derives its origin from the word signifying bow. This custom dates from the most remote antiquity—from the time when men earned their means of subsistence by the bow. It is almost universally prevalent at the present day among the most primitive and savage tribes; and to this custom we owe our knowledge of one of the most powerful poisons that exists. We find frequent allusions to it in the classical writers.

Homer represents Ulysses as sending to Ephyra for poison wherewith to anoint his arrows. The story of Hercules dipping his arrows in the venom of Lernæan hydra, their deadly effects, and the dreadful accident which befel Chiron and Philoctetes, are familiar to you all. This story is an indication of the nature of the poison employed; and we have it on the authority of numerous writers, that snake poison was frequently used for this purpose. Such was the custom of the ancient Scythians, who likewise mixed the venom with human blood, of itself regarded as a virulent poison, in order to intensify the effects. Even at that early period, they had discovered the fact, though its explanation by the physiological relation between absorption and excretion is only of recent date, that, when taken naturally, the poison was inocuous, and was only fatal when directly introduced into the blood by means of a wound. Pliny makes a curious observation in reference to this practice. He looks upon it as a mark of the depravity of mankind; for what other animal (he asks) but man poisons his weapons? Ælian, however, is of opinion that man in this respect has only followed the bad example of the wasps: "for," said he, "the wasps, when they see a dead snake, alight on it, and dip their stings in its venom"—a curious fact in natural history.

Another very ancient use of poisons was for the purpose of the *ordeal*—a method of judicial investigation which also obtains among many tribes at the present day. With this custom we generally associate the physostigma or ordeal bean of Old Calabar. It is supposed that it was a poison of some kind which constituted the *maar* or *bitter water*, of which we read in the book of Numbers, and which was used as a judicial test of the honor of wives accused of infidelity by jealous husbands. Harmless to one who was innocent, the effects in the case of a guilty woman were dire enough, causing the belly to swell and the thigh to rot. It is very doubtful whether this were really a poison. It is more likely that the dreadful invocations of the imposing preliminary ceremony were sufficient to deter any but an innocent woman from draining the contents of the cup.

One of the most curious chapters in the literature of poisoning is that which relates to the use of poisonous substances in the preparation of *philtres*, or love-potions, which were administered under the idea that the affections of the person so practised on would be gained by the individual who employed them. Though the means adopted were often ridiculous enough, and partook more of the character of sorcery and incantation, yet in many cases they were not of the same harmless nature, and the evil effects which frequently resulted assumed such an importance in the eye of the law, that, even at an early period, the administration of such filtres was looked upon in the same light as more serious attempts of poisoning, and was punished as a capital offence. The idea involved in the use of philtres is no doubt, to a certain extent, based on sound physiological principles, and was properly suggested by an attentive observation on the habits of the lower animals; and it still leads to crime, for we yet occasionally meet with cases in which poisonous substances are employed for a similar purpose by vile and deluded miscreants. There is great difficulty in ascertaining the true nature of many of the substances to which the ancients ascribed such virtues, and in estimating how much truth there is in the narratives which have come down to us.

Many learned commentaries have been written on the *dudain*, or mandrakes, which were held by the Jews of old in high repute as an aphrodisiac, and also as a cure for sterility—an opinion strengthened by the results which apparently followed its use in Rachel's case. Probably the inquiries have been stimulated by the desire to rediscover a remedy which would be of such immense value to obstetricians. The most likely of the suppositions which may have been advanced respecting this plant is, that it belonged to the natural order of the *Atropaceœa.* This opinion would accord best with the descriptions which we read elsewhere of the effects which resulted from the administration of philtres, viz.: a kind of frenzied delirium, such as might be produced by some one of the deliriant narcotics belonging to this group. In all probability, it is to some such plant, in its connections with philtres, that Shakespeare refers when he speaks of

> "The insane root
> Which takes the reason prisoner."

Among the Greeks and Romans, Medea was universally regarded as the greatest adept in the art of preparing philtres; and hence the term "*Medeides herbæ*" was used by Horace and Ovid to designate such substances generally. Next in reputation stand the Thessalian women, who may be supposed to have learned the art from Medea, and who attained great celebrity in all that related to poisoning, sorcery and incantation. Hence "Thessalian arts" and

"Thessalian poisons" are frequently employed by the classical writers as generic terms, applicable to all forms of poisoning.

Philtres are described by Ovid and others as affecting the reason and causing a phrensy, which sometimes terminated fatally.

"Philtra nocent animis, vimque furoris habent."

Lucretius, the great philosophical poet of the Ciceronian era, is said to have written his poem "On the Nature of Things" in the intervals of delirium occasioned by a philtre or love-potion secretly administered to him by his wife or mistress, Lucilia; and Lucullus, the Roman general, is stated to have died in a state of delirium from a similar cause. The laws of the Twelve Tables contained special provisions against this form of poisoning. It was included under the head of sorcery, and was punished as a capital offence: "*Qui malum carmen incantat, malum venenum faxit duitve, parricida esto.*"

Though it would appear, therefore, both from the accounts we have received and from the necessity of special enactments against it, that the administration of philtres often led to results far more serious than were contemplated by those who used them, we can hardly regard otherwise than as a subject of amusement the credulity which prevailed even to a very late period with respect to many substances, held in high repute, and supposed to be possessed of marvelous properties. Most of these substances would hardly come under the head of poisons, or even noxious substances, as we understand them; but yet in most countries their administration was forbidden, as coming under the head of sorcery and witchcraft.

The idea which guided the selection of some of the substances regarded as stimulants of love we might understand, though it would be rather difficult to do so in the case of others. One of the most celebrated agents of this sort is the substance called *hippomane*, described by some as a kind of *vernix caseoso* found on the head of a newly born foal; by others, as of a much more disgusting nature (*"virus destillans ab inguine equæ coitum maris appetentis."*) Animals of various kinds, and parts of certain animals, such as the testicles, the hair from the tip of a wolf's tail, a bone from the left side of a toad devoured by ants, ribs of snakes, etc., were all used as powerful ingredients in love cups. Menstrual blood, and particularly that of a red-haired woman, was not only greatly esteemed as a philtre, but was regarded as a most powerful poison. It is a curious circumstance, and one which we found great difficulty in accounting for, that not merely human blood, but the blood of animals in general, was universally looked upon as a poison in ancient times; and that this belief should have been shared by medical writers up to a comparatively recent period. We read in Herodotus that Psammenitus, King of Egypt, was put to death by Cambyses by being made to drink bullock's blood. Such also, according to the popular belief, was the mode in which Themistocles committed suicide. Unwilling to fight against his own countrymen, he drank a goblet of the blood of a sacrificial ox, and expired almost immediately. Even up to the time of Blumenbach, in the middle of the last century, the belief that blood was poisonous was general, and it was treated of as such in many learned works on poisons and legal medicine. Zacutus Lusitanus, writing in 1657, relates many instances of dreadful effects resulting from the drinking of blood. It is worth while quoting one of these. A student, animated by the spirit of practical joking, gave to another, instead of wine, two ounces of the blood of a red-haired woman mixed with sugar. The victim of this joke did not feel any immediate bad effects, but after a day or two he passed into a raving state, and became ultimately a confirmed idiot. One is tempted to remark that the individual in question cannot at best have been far removed from this state, if he could mistake for wine the nauseous mixture offered to him. And in the opinion of Andreas Cæsalpinus, who nearly anticipated Harvey in the discovery of the circulation, it is to human blood that we owe the origin of one of the most virulent contagious diseases. He traces the origin of syphilis to the fact that the Spaniards, in abandoning the small town of Somma, at the foot of Mount Vesuvius, mixed all the wine of the place with the blood of the patients in the hospital of St. Lazarus.

Blumenbach, in his lectures, recommended his students to make experiments in order to clear up all doubts as to the poisonous qualities of blood. One student experimented on himself, and drank seven ounces of warm bullock's blood without experiencing any evil effects. Another preferred to experiment on a dog, which likewise survived the experiment.

Returning from this digression on blood once more to the subject of philtres, we find that the belief in them was strong even as late as the time of Van Helmont, in the middle of the seventeenth century. Van Helmont himself was fully persuaded of their efficacy, and perhaps the most remarkable statement in regard to them occurs in one of his writings. Here is a translation of it: "I know a plant of common occurrence, which if you rub and cherish in the hand till it becomes warm, and then take the hand of another and hold it till it also becomes warm, that person will forthwith be stimulated with love for you, and continue so for several days."

A plant is mentioned by Theophrastus, to which even more powerful effects were ascribed, but which need not be more particularly specified.

There were not a few, however, even in ancient times, who were skeptical as to philtres. Ovid, who speaks so much of them, had his doubts; and, in a letter to a young lady, he recommends, as far the most preferable prescription, "*ut ameris, amabilis esto.*"

From these and similar truths, the influence of philtres gradually ceased to be believed in, and disappeared from medical literature by the middle of the eighteenth century. Closely connected with the practice of brewing love-drinks was the still more pernicious and dangerous one of dealing in abortifacients. What we now call the crime of abortion was not in the early times regarded as such. False political and social doctrines had much to do with the prevalence of the practice before the dawn of Christianity, and it was not till the time of the early Christian emperors that legislative measures were enacted to check it. It was the custom in early ages not to regard a newly born child as a human being, whose life was worthy of preservation, till it had been taken up by the father and placed in the mother's bosom. It was at the option of the father whether the child should be exposed or reared. Infanticide, therefore, was looked upon as a regular social institution. Of much less import did it seem to kill a child in its mother's womb, or to cause its expulsion at a time when its death would be certain. The politics of Plato and Aristotle did much to encourage the practice; for they held, as do the followers of Malthus at the present day, that the number of children born every year in a State should be regulated and restricted; and therefore they recommended abortion as a means of checking over population. This license was, of course, a premium on immorality; and the premium reached its highest extent in the days of the Roman empire, as the pictures drawn by Juvenal sufficiently show. The morals of the Roman matrons at that time were such as to lead them to resort to the practice on the most frivolous motives. They had an abhorrence of everything which they thought might spoil their beauty or destroy their symmetry of form. They looked upon a pregnant woman as an "*indecens onus*," and had recourse to abortifacients "*ne rugis ventrem Lucina notaret.*" It is accounted by Seneca as one of the virtues of his mother, Helvia, that she had never been guilty of this debasing vice. The agents who pandered to the licentiousness and vice of this period were numerous, and well-skilled in the knowledge of almost all the mechanical and oxytocic means of procuring abortion with which we are at the present day acquainted. Then, as now, the attempts were frequently followed by fatal consequences.

Though the practice of abortion was not usually considered criminal, and at the worst only visited by moral censure, there were occasions in which it was held as felony. Cicero relates a case where a woman was condemned to death for having, in collusion with the heirs presumptive, practised abortion on herself and thus defrauded her husband of children.—*Chemist and Druggist.*

